# Pembrolizumab-Induced Autoimmune Grade 4 Neutropenia in a Patient With Advanced Bladder Cancer: A Case Report

**DOI:** 10.7759/cureus.31552

**Published:** 2022-11-15

**Authors:** Mafalda Miranda Baleiras, Carolina Vasques, Marta Pinto, Helena Miranda, Ana Martins

**Affiliations:** 1 Medical Oncology, Centro Hospitalar de Lisboa Ocidental, Lisbon, PRT; 2 Medical Oncology, Centro Hospitalar Tondela-Viseu, Viseu, PRT

**Keywords:** bladder cancer, pembrolizumab, immune-related adverse effects, neutropenia, immune-checkpoint inhibitors

## Abstract

Neutropenia is amongst the rare, but potentially life-threatening complications of immune checkpoint inhibitors (ICI). Awareness about this dangerous toxicity and its adequate treatment since early detection is of utmost importance. Unfortunately, there are no therapeutical guidelines to deal with neutropenia specifically. The best alternative is informed extrapolations based on reported neutropenia cases and established guidelines for other immune-related adverse events. We report a case of pembrolizumab-related grade 4 neutropenia in a patient with metastatic bladder cancer. She was successfully treated with immunosuppressive and supportive measures. Further studies are required to understand the range of immune-related adverse events and to improve their management.

## Introduction

Immune checkpoint inhibitors (ICI) have revolutionized the therapeutic management and prognosis of several cancers. By blocking co-inhibitory immune checkpoint molecules, such as programmed cell death receptor-1 or ligand-1 (PD-1/PD-L1), ICI promotes antitumor T-cell responses.

Compared to cytotoxic chemotherapy, immunotherapy is better tolerated. However, the loss of self-tolerance can lead to autoimmune-like events, commonly labelled as immune-related adverse events (irAEs). They can harm virtually any organ [1].

Haematological irAEs (haem-irAEs) are rare, occurring in up to 1% of irAE cases [2]. Neutropenia, autoimmune haemolytic anaemia, and immune thrombocytopenia were the most common types of haem-irAE during PD-1/PD-L1 treatment in patients registered in three French pharmacovigilance databases [3,4]. Despite their rarity, haem-irAEs are potentially life-threatening events with a mortality rate of 2%-14% [1]. To date, no clear treatment recommendations exist for immune-related neutropenia (irNeut). Hence, early recognition and appropriate patient management are important.

In the absence of sound guidelines, smart extrapolation from case reports combined with established recommendations for other irAES provides an alternative toolkit to guide the treatment of irNeut. This may help to secure timely and safe patient care. Herein, we present a rare case of a grade 4 neutropenia induced by pembrolizumab, an anti-PD-1 antibody.

## Case presentation

A 59-year-old Caucasian female, with a 40-pack-year smoking history, presented with a 12-month history of painless intermittent gross haematuria and progressive asthenia. She reported no other systemic symptoms. Her past medical history was unremarkable. Family history of immune-related diseases or malignancy was denied. She had no use of dietary or herbal supplements. 

General examination was unremarkable. A computed tomography (CT) scan of the chest, abdomen, and pelvis showed multiple lung lesions suggestive of metastasis and an irregular nodule involving the left wall of the bladder, with a maximum cross-sectional size of 26 x 19 mm. The patient underwent transurethral resection of the bladder tumour, which revealed a necrotic mass involving the anterior and left side of the bladder wall. Anatomopathological analysis was consistent with high-grade urothelial carcinoma with invasion of muscularis propria. Therefore, the patient was diagnosed with metastatic bladder cancer.

**Figure 1:**
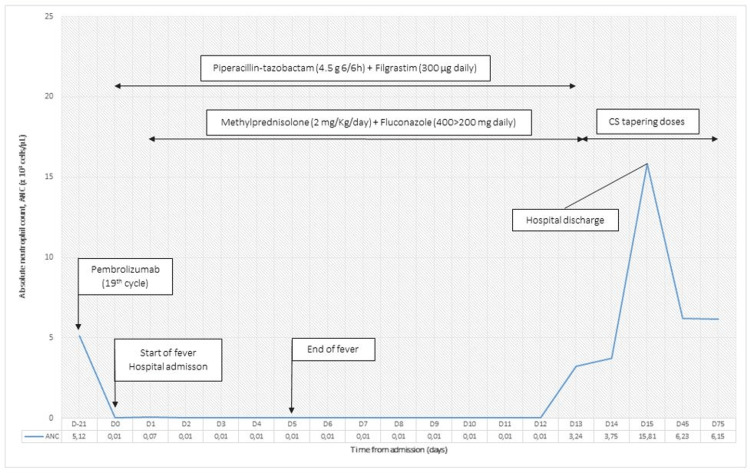
Treatment course and absolute neutrophil count over time Therapeutics received and major events are described along with their timeline. CS: corticosteroids.

She was initially treated with palliative chemotherapy: cisplatin (70 mg/m2, day 1) and gemcitabine (1000 mg/m2, days 1 and 8), every three weeks. After the third cycle, a whole-body enhanced CT scan was conducted which revealed greater left bladder tumour infiltration (maximum cross-sectional size of 63 x 50 mm). Upon disease progression, she was then started on single agent pembrolizumab (200 mg), every three weeks. Her baseline blood count before starting immunotherapy was all within the normal range (Figure 1). No major toxicities were reported during the course of pembrolizumab.

Three weeks after completing the nineteen infusions of pembrolizumab, she presented to the emergency room with fever, chills and odynophagia. On examination, she had grade 3 hypotension (73/48 mmHg) and grade 3 oral mucositis (according to Common Terminology Criteria for Adverse Events (CTCAE) version 5). Laboratory investigation revealed an absolute neutrophil count (ANC) of 10 cells/μL and a raised serum C-reactive protein of 26 mg/dL. Haemoglobin was 12.8 g/dl, and platelet count was normal (160 000 platelets/mcL). The patient was hospitalized with grade 4 febrile neutropenia and started on piperacillin-tazobactam (4.5 g every six hours), filgrastim (300 µg daily) and intravenous vigorous fluid therapy. All her infectious workup, including blood cultures and imaging, were negative. Confounders, such as folate and vitamin B12 deficiency or medications that cause neutropenia (metamizole, clozapine and sulfasalazine) were also ruled out. Intravenous methylprednisolone (2 mg/Kg/day) was initiated on day two, along with fluconazole (loading dose of 400 mg followed by 200 mg daily). Pembrolizumab was permanently suspended.

Thirteen days after admission, her neutrophil count recovered with an ANC of 3240 cells/μL. Therefore, piperacillin-tazobactam and filgrastim were suspended and methylprednisolone was converted to prednisolone (150 mg daily, per os). She was discharged from the hospital on day fifteen. Her prednisone was tapered weekly over the next two months. She experienced no recurrence of neutropenia and had stable disease on follow-up CT two months later. Her case is still being closely monitored.

## Discussion

Immune evasion is a hallmark of cancer. Immune checkpoints (IC) are inhibitory regulators of the immune system [5]. They play a vital role in maintaining immune self-tolerance and controlling the duration and extent of immune responses in order to prevent autoimmune reactions. Cancer cells engage these inhibitory immune checkpoint pathways in order to escape and proliferate [5].

Blockade of key suppressive IC pathways through ICI is able to restore the ability of the immune system to mount an effective anti-tumour response. Monoclonal antibodies targeting the immune regulatory checkpoint receptors of PD-1 (through pembrolizumab, nivolumab, cemiplimab and dostarlimab), PD-L1 (through atezolizumab, avelumab and durvalumab) and cytotoxic T-lymphocyte-associated protein-4 (CTLA-4; through ipilimumab) have improved clinical outcomes in a variety of malignancies. Despite these benefits, ICI carries a unique spectrum of toxicities related to an exaggerated immune response against self-tissues, termed irAEs [5].

Although any organ can be affected, irAEs most commonly involve the skin, the gastrointestinal tract, the liver and endocrine glands [4]. The incidence, onset and severity of irAEs depend on the class and dose of administered ICI, underlying malignancy and patients’ characteristics [6,7]. They can happen anytime during the treatment course and even after completion [8]. Dupont et al. (2019) pointed out that these events may occur in about 50% of patients treated with anti-PD-1 monotherapy, leading to therapeutic discontinuation in approximately 10% of them [5].

With an overall reported incidence of 1%, haem-irAEs comprise a variety of entities. Petrelli et al. (2018) conducted a systematic review and meta-analysis of trials of PD-(L)1 inhibitors in cancer patients. Among 9324 patients, anti-PD-(L)1 agents were associated with a moderate risk of anaemia (10%) and a low risk of neutropenia and thrombocytopenia (0.9% and 2.8%, respectively), with negligible risk of febrile neutropenia (0.45%). Incidence of all grades and G3-5 neutropenia was 0.94% and 1.07%, respectively [2].

In a more recent analysis, based on data from 168 haem-irAEs observed in the World Health Organization’s pharmacovigilance database, the VigiBase, autoimmune hemolytic anaemia was the most commonly reported hematologic toxicity, followed by immune thrombocytopenic purpura [3]. Toxicities related to anti-CTLA-4-based therapy occurred earlier than those associated with anti-PD-(L)1 inhibitors (median 23 vs. 47.5 days) [3]. In this publication, concurrent nonhematologic irAEs occurred in 23% of these patients. Our patient, however, presented with isolated neutropenia with no other accompanying irAEs.

The median onset of ICI-induced neutropenia is 10 to 11 weeks, with a median duration (at grade 2 or worse) of 13 to 16.5 days [1]. In our case, the onset of grade 4 irNeut was later (57 weeks after) and the median duration was similar (13 days).

IrNeut can lead to significant morbidity and mortality arising from bacterial or fungal infection. Based on VigiBase, 67% of isolated neutropenia were associated with fever, which corresponded to our patient’s presentation. In this database, the mortality rate from septic shock during the episode of febrile neutropenia was 11% [4].

Robust guidelines on the management of ICI-induced neutropenia are missing. Yet, timely diagnosis and care are decisive because of its high mortality rate. The second-best anchor to deal with ICI-induced neutropenia lies with standardized approaches for irAEs management [9]. To mitigate infection risk, granulocyte colony-stimulating factors (G-CSF) should be used until the resolution of neutropenia [4,7,10]. And, in cases of febrile neutropenia, empiric broad-spectrum antibiotics should be promptly administered. Despite some conflicting data regarding the use of systemic corticosteroids due to their potential of increasing the risk of infection, steroid therapy has been reported as part of the initial management of irNeut [4,7,10,11]. In our clinical case, the early introduction of antibiotic G-CSF and corticoid may have contributed to the patient’s satisfactory evolution. The successful corticoid tap off without recurrent neutropenia events after stopping the ICI is also worth mentioning.

## Conclusions

To sum up, the risk of infection makes irNeut a life-threatening disease. The increasing number of cancer patients going through ICI therapy raises the absolute incidence of lesser-known irAEs. A coordinated multidisciplinary approach is essential for early diagnosis and timely treatment to improve patient care. ICI discontinuation should be strongly recommended. Prompt intervention based on immunosuppressive and supportive measures perhaps mitigates irNeut duration and severity, thus preventing potentially fatal outcomes. These patients should therefore be closely monitored.
